# Long-term dynamics of plant communities after biological remediation of oil-contaminated soils in far north

**DOI:** 10.1038/s41598-021-84226-5

**Published:** 2021-03-01

**Authors:** A. B. Novakovskiy, V. A. Kanev, M. Y. Markarova

**Affiliations:** 1 Institute of Biology Komi SC UB RAS, Kommunisticheskaya st., 28, Syktyvkar, Russia; 2 Federal Scientific Vegetable Center, Selektsionnaya st. 14, Odintsovo District, Moscow Region, Russia

**Keywords:** Restoration ecology, Environmental impact, Abiotic

## Abstract

We studied the long-term dynamics of plant communities after bio and phytoremediation of oil-polluted soils. Nine plots located in European Northeast and treated using various bioremediation methods were monitored from 2002 to 2014. Geobotanical descriptions (relevés) of each plot were performed in 2006 and 2014, and Grime’s theoretical CSR (competition–stress–ruderality) framework was used to assess the vegetation state and dynamics. We observed a clear shift of communities from pioneer (where ruderal species were prevalent) to stable (where competitor species were dominant) states. However, the remediation type did not significantly impact the vegetation recovery rate. After 12 years, all methods led to a 55–90% decrease in the oil content of the soil and a recovery of the vegetation cover. The plant communities contained mainly cereals and sedges which significantly differed from the original tundra communities before the oil spill. The control plot, treated only by mechanical cleaning, had minimum oil degradation rate (50%) and vegetation recovery rates, although, in CSR terms, its vegetation assemblage resembled the background community. Cereals (*Agrostis gigantea*, *Deschampsia cespitosa*, *Phalaris arundinacea*, and *Poa pratensis*), sedges (*Carex canescens*, *Carex limosa*, and *Eriophorum vaginatum*), and shrubs (Salix) were found to be the most effective species for phytoremediation, exhibiting high community productivity under the harsh northern conditions.

## Introduction

Modern human activity is strongly associated with the extraction, storage, transportation, and refining of large volumes of petroleum products. During the operation of oil production facilities, accidents such as spills and leaks of various magnitudes are possible. These lead to the pollution of large areas of the earth's surface with oil and oil products, and the attendant negative impacts on local flora and fauna. The adverse effects on vegetation include seed germination suppression, reduction in the number of photosynthetic pigments, slower nutrient absorption, and decrease in the size of plant organs (roots, leaves, and stems)^1–4^. Large oil spills can have catastrophic effects on vegetation, sometimes leading to its complete extinction^5–9^.

Technological development for fast and high-quality remediation of soil, water, and vegetation is one of the most challenging endeavors of modern ecological science. To reduce the oil concentration in soil and water, various physical^10^, chemical^11–14^, and biological methods are employed. Biological methods can be divided into two large groups: bioaugmentation and phytoremediation. The first one involves biological preparations based on natural oil-oxidizing microorganisms^15–22^. The second one employs the use of vascular plants for restoring soil and soil fertility^1,4,23–32^.

Remediation methods differ in their energy-cost, technological complexity, and efficiency^33^. Among all methods, biological methods have a good trade-off between efficiency and cost^22^. They do not require special technical skills^34^ and generally do not negatively impact ecosystems^22,33,35^.

Biological methods require the selection of appropriate microorganisms, algae, fungi, or plant species, which show a high oil degradation rate and produce large amounts of biomass. Due to the complex relationships within ecosystems, it is important to test selected species, both in the laboratory and under field conditions^36,37^. Field studies are particularly relevant for the far north and Arctic regions. Although these regions have vast hydrocarbon reserves, the Arctic flora and fauna are particularly vulnerable to external influences^38,39^. In those harsh climatic conditions, even the smallest impact on nature can have a significant destructive effect.

Usually, scientific studies devoted to the recovery of oil-contaminated territories focus on the oil decomposition rate and the speed of vegetation biomass growth^26,29,40,41^. However, the issues of long-term dynamics of restored communities and their affinity to the background vegetation is also important^7,42^. Vegetation analysis is a convenient tool for assessing the quality of ecosystem restoration and reflects the state of the soil and ecosystems in general.

The vegetation state and its dynamics were assessed by employing Grime’s theoretical CSR (competition–stress–ruderality) framework^43^. This theory divides plant species into three types, based on their responses to stress and disturbance^44,45^. They both inhibit ecosystem production but different way. Stress is a long-term factor usually imposed by ecological conditions (lack of light, water, mineral nutrients, suboptimal temperatures). Disturbance is a short-term factor which restricts the biomass by destroying it. It usually arises from animal grazing, trampling, mowing, soil erosion or other physical impacts.

The competitors grow under productive conditions (low levels of stress and disturbance). These species usually have high canopy levels and large leaves, stems, and roots which allow them to efficiently capture resources. Stress-tolerant species are typical for habitats with chronically unproductive conditions (high stress and low disturbance). Usually these are small species with long life-span, slow growth rate, and rhizome reproduction. Ruderals predominate in productive habitats with weakened competition between species due to mechanical impacts on vegetation (low stress and high disturbance). These plants are characterized by a high growth rate, prevailing seeds reproduction, and a short lifespan. These are often spring ephemeroids or weeds.

The ratio of the species of the different CSR types allows for the assessment of an integral characteristic of the vegetation. Thus, the predominance of competitive species is often associated with a low anthropogenic impact (abandonment) and favorable environmental conditions. The presence of a large number of stress-tolerant species is associated with adverse environmental factors. Ruderal species are associated with land cultivation, grazing, and other forms of disturbances^46–48^.

The aim of this study was to assess the long-term effect of different ecosystem remediation methods in European northeast environmental conditions after an accidental oil spill in terms of vegetation restoration characteristics.

## Materials and methods

The experiments were conducted at the Verkhnevozeyskoye oil field (Usinsky district, Komi Republic: 66°37′40″ N, 57°07′56″ E), in the Lukoil Usinskneftegaz area, where oil spills occurred several times between 1989 and 1996^39^. This territory belongs to the forest-tundra zone and is characterized by harsh climatic conditions: low temperatures, strong winds, and a short growing season^49^.

The contaminated area of the experimental site was approximately 2 ha and located on a high peat bog with a peat deposit thickness of up to 1.5–2.0 m, in which the oil penetrated to a depth of 1.0 to 1.5 m. After technical recultivation (removing of surface oil) and land melioration (removal of excess water and oil from the soil by drainage), the site was plowed to a depth of 25–30 cm and then divided into test plots of 0.2 ha each (40 m × 50 m).

The experiments for assessing the efficiency of the various bioremediation methods (biopreparations, sorbents, and agricultural techniques) started in June 2002 (Table 1). In the beginning, the soil moisture ranged from 65 to 88%, while the oil concentration ranged from 150 to 450 mg g^−1^ ^39^. The oil concentration of the soil was determined by fluorimetric method using a Fluorat-02 fluid analyzer (Russia)^50^.

**Table 1:** Brief description of the remediation methods^39^.

Plot	Bio-recultivation method	Seeded plants, fertilizers
1	Biopreparation “Petrolan”^1^	*Phalaris arundinacea*, *Agrostis gigantea*, *Phleum pratense*, *Trifolium pratense*, *Avena sativa*. Mineral fertilizers^9^
2	Control	Only mechanical oil removal. No seed plants, fertilizing, or biopreparations
3	Biopreparation “Inipol EAP 22”^2^	*Phleum pratense*, *Agrostis gigantea*, *Deschampsia cespitosa*. Mineral fertilizers
4	Biopreparation “Universal” ^3^	*Phleum pratense*, *Agrostis gigantea*, *Avena sativa*. Mineral fertilizers
5	Biopreparation “Omug”^4^	*Phleum pratense*, *Avena sativa*. Mineral and organic fertilizers
6	Biopreparation “Universal”, lignin sorbents^5^, BAG^6^	*Deschampsia cespitosa*. Compost and mineral fertilizers
7	Phytoremediation (without biopreparation)	*Avena sativa*, *Phleum pratense*. Mineral fertilizers, dolomitic meal
8	Biopreparation “DEKONTAM-3” ^7^	*Phleum pratense*, *Avena sativa*. Compost, lime, and mineral fertilizers
9	Biopreparation “Roder”^8^	*Phalaris arundinacea*, *Phleum pratense*, *Avena sativa*. Mineral fertilizers and lime

^1^“Petrolan” Design—commercial confidentiality. Developer—“Priborservis”, Tomsk, Russia.

^2^“Inipol EAP 22”. Design—commercial confidentiality. Developer—“TotalFinaElf”, France.

^3^“Universal”. Design—yeasts *Rhodotorula glutinis* and bacteria *Rhodococcus egvi, Rhodococcus erythropolis, Pseudomonas fluorescens*. Developer—Institute of biology Komi Science Center, Syktyvkar, Russia.

^4^“Omug”. Design—oil-destructing microorganisms *Bacillus subtilis* and bacteria *Rhodococcus* sp. deposited on the organic fertilize surface. Developer—“Nika”, St. Petersburg, Russia.

^5^Lignin sorbents—dried and crushed compost with embedded oil-destructing microorganisms isolated from the oil-contaminated soils of the study area^51^.

^6^BAG—Biologically Active fertilizer-seeding granules on the compost basis with fertilizers and seeds of perennial herbs^52^.

^7^“DEKONTAM-3”. Design—commercial confidentiality. Developer—«Dekonta a.s.», Czech Republic.

^8^“Roder”. Design—bacteria *Rhodococcus ruber* и *Rhodococcus erythropolis*. Developer—Chemical Faculty of Moscow State University, Moscow, Russia^18,53,54^.

^9^Mineral fertilizers—ANP (ammonium nitrate phosphate) fertilizer at a rate of 350 kg/ha. Fertilizer was introduced in 2002. Additional feeding was not carried out.

During the next few years, the soil moisture changed unevenly due to the fragmented siltation of the drainage channels. This caused variations in the distribution pattern of the cultivated and invading native plants. Mineral fertilizers, introduced in 2002, ensured the start of growth of sown plants and the efficient oxidation of the oil by microorganisms. Further feeding was not carried out. The development of the sown and invading plants was determined by the soil moisture level and the nature of the residual pollution which correlated with the amount of the oil-destructing bacteria. Soil contamination was uneven and varied greatly, even within the same plot (Table 2).

**Table 2:** Oil concentration in soil (mg g^−1^); 0–20 cm depth (9 collected samples per plot).

Plot	Year (Average values ± SD)			Wilcoxon test (2002–2006)		Wilcoxon test (2006–2014)	
	2002	2006	2014	W	p-value	W	p-value
1	216.1 ± 25.3	72.2 ± 32.5	37.2 ± 18.9	20	**0.02**	15	0.25
2 (control)	204.4 ± 35.0	176.1 ± 34.3	99.4 ± 17.9	17	0.10	19	**0.03**
3	199.4 ± 45.5	96.7 ± 38.2	91.7 ± 34.6	20	**0.02**	13.5	0.46
4	292.2 ± 40.3	83.3 ± 30.8	38.3 ± 25.2	20	**0.02**	17	0.11
5	243.9 ± 73.7	151.1 ± 73.1	110.0 ± 39.5	16	0.18	14	0.39
6	319.4 ± 58.0	83.9 ± 28.5	26.1 ± 21.6	20	**0.02**	20	**0.02**
7	228.9 ± 37.9	105 ± 11.7	65.6 ± 17.6	20	**0.02**	20	**0.02**
8	363.3 ± 66.1	191.7 ± 51.8	125 ± 23.3	20	**0.02**	17	0.10
9	305.6 ± 64.6	123.3 ± 47.8	30.6 ± 20.1	20	**0.02**	18	0.07
Reference plot	3.0 ± 0.1	2.9 ± 0.1	2.9 ± 0.1	11.5	0.80	9	0.90

SD denotes standard deviation; significant differences (p < 0.05) are bold.

Two relevés were performed for the testing plots, four and twelve years into the experiment (in 2006 and 2014, respectively), using the standard geobotanical methods. We focused on the vascular plants which were the predominant species in the plots. The species nomenclature used hereafter is in accordance with www.theplantlist.org.

We selected a willow-dwarf birch sedge-horsetail swamp as the reference plot. This was located on a site with a similar to experimental plots landscape, away from the oil-contaminated area. The most abundant species in the swamp were *Salix myrtilloides*, *S. lapponum*, *Betula nana*, *Equisetum fluviatile*, *Menyanthes trifoliata*, *Comarum palustre*, *Andromeda polifolia*, *Eriophorum vaginatum*, and *Calamagrostis lapponica*. The moss-lichen layer consisted mainly of sphagnum mosses (*Sphagnum magellanicum* and *S. fuscum*), with small inclusions of *Polytrichum commune*. The water content of the reference plot was 80–89%.

Full relevés for the experimental and reference plots are provided in the supplementary materials (Suppl. 1).

The integral position of relevés in the CSR triangle was estimated using the community-weighted mean (CWM) method^55^. To reduce the influence of dominant species on the resulting scores, we used the logarithmic transformation ln (*A* + 1), where *A* denotes the species abundance^56^. The CSR species types were determined according to J.G. Hodgson^57^. Furthermore, each CSR type was presented in tertiary coordinates form from 0 to 100%, where 0 signifies the absence of traits of this type, and 100% is a clearly defined strategy^58^. The CWM score is defined as follows:

1 $$ CWM = \frac{{\sum_{i = 1}^{N} {\ln (A_{i} + 1)*B_{i} } }}{{\sum_{i = 1}^{N} {\ln (A_{i} + 1)} }}, $$

where *A*_*i*_ is the abundance of the *i*-th species in the relevés, *B*_*i*_ is the score value along the C, S, R axes of the Grime’s triangle for the *i*-th species, and *N* is the number of species.

The species diversity of the relevés was estimated based on the number of recorded species and the Shannon species diversity index^59,60^, which is expressed as follows:

2 $$ H = - \sum\limits_{i = 1}^{N} {p_{i} *\log_{2} (p_{i} )}, $$

where *H* is the Shannon index, *p*_*i*_ is the contribution of the *i*-th species to the plant community (the ratio of a species abundance to the sum abundances of all species of a given community), and *N* is the number of species.

Euclidean distances were used to measure the difference between the positions of the communities in the CSR triangle. A statistical analysis was carried out using the non-parametric Wilcoxon rank tests and ordination by using the non-metric multidimensional scaling method (NMDS)^61,62^. Calculations were performed with the program R v.3.5.2. with “Vegan” package.

## Results

The oil content of the soil in the treated plots decreased from 200–360 mg g^−1^ in 2002 to 30–125 mg g^−1^ in 2014. This decrease occurred unevenly over time. Over the first period (2002–2006), statistically significant changes were observed in all the experimental plots, except plot 5 and the control. During the second period (2006–2014), the oil content significantly decreased in the control and only for two of the treated plots (Table 2).

**Figure 1:**
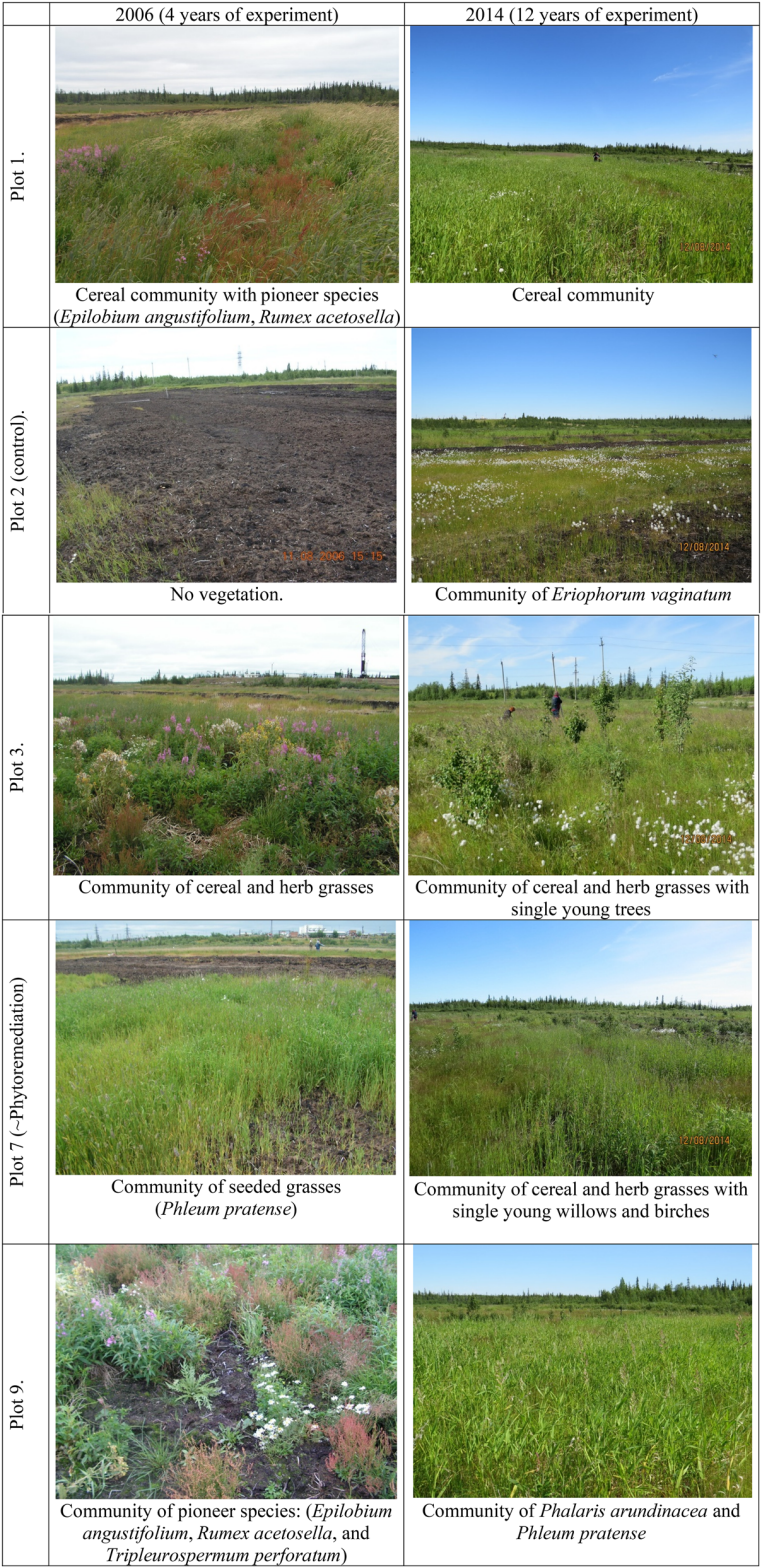
Vegetation changes at the experimental plots.

By the fourth year of the experiment (2006), the cereals seeded during the remediation (*Agrostis gigantea*, *Deschampsia cespitosa*, *Phalaris arundinacea,* and *Phleum pratense*) became dominant. Also, the presence of a single species, which was not used in remediation, was noticed. These are intermediate CSR species (*Rumex acetosella* and *Stellaria graminea*) and ruderal types (*Lapsana communis, Rumex crispus, Plantago major,* and *Tripleurospermum perforatum*) (Fig. 1). In 2006, the total projective cover at the experimental plots was 70–80% (Table 3).

**Table 3:** Biodiversity indicators for experimental, control, and background territories.

	Projective cover (%)	Number of vascular species	Shannon index (H)	Euclidian distance between CSR scores of 2006 and 2014
Plot 1	70–80/70–80	21/16	2.1/1.9	28.8
Plot 3	60–70/50–60	22/17	3.5/3.2	16.4
Plot 4	50–60/45–50	21/10	3.7/1.5	33.4
Plot 5	60–70/70–75	15/13	1.7/2.2	18.9
Plot 6	90–95/80–85	16/6	1.4/0.5	18.2
Plot 7	80–90/70–75	15/12	1.7/1.5	13.4
Plot 8	50–60/40–45	16/13	2.8/2.0	15.9
Plot 9	70–80/70–80	19/22	2.5/2.9	15.0
Plot 2 (Control)	No vegetation/25–30	0/17	–/3.3	–
Reference plot	Did not measure the projective cover/55–60	16/10	–/2.0	–

First value in the cells—year 2006/second value—year 2014. The dash symbol “-” denotes absent or incomplete data.

By 2014, four of the six species used for remediation had retained their presence. The *Phleum pratense* (CSR type according to Grime’s classification) was found to be less abundant; *Agrostis gigantea* (CR type) was more abundant. Other seeded cereals: *Deschampsia cespitosa* (CS/CSR) and *Phalaris arundinacea* (C) were as abundant as at the beginning of the experiment.

**Figure 2:**
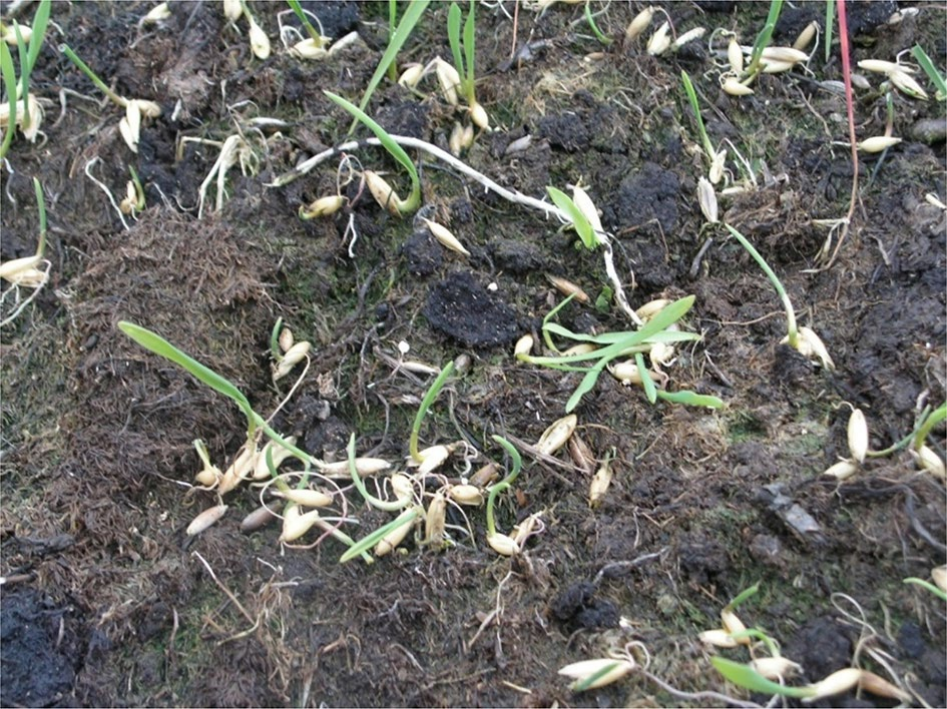
Oats (*Avena sativa*) in seedling form. Plot 4 (2006).

Two species, *Avena sativa* (−) and *Trifolium pratense* (CSR) had the lowest survival rate. *Avena sativa* was sown on six out of nine experimental plots. In 2006, this species was observed in seedling form only on 3 plots (4, 8, and 9; Fig. 2), with low biomass and abundance. In 2014, *Avena sativa* was not detected on any of the plots. Meanwhile, *Trifolium pratense* had a reduced presence in 2006, disappearing completely by 2014.

By 2014, there was almost complete disappearance of ruderal species. These included the pioneer plants: *Lapsana communis* (R/CR type), *Tripleurospermum perforatum* (R), *Polygonum aviculare* (R), and the weeds: *Leucanthemum vulgare* (CR/CSR), *Artemisia vulgaris* (C/CR), and *Plantago major* (R/CSR).

**Figure 3:**
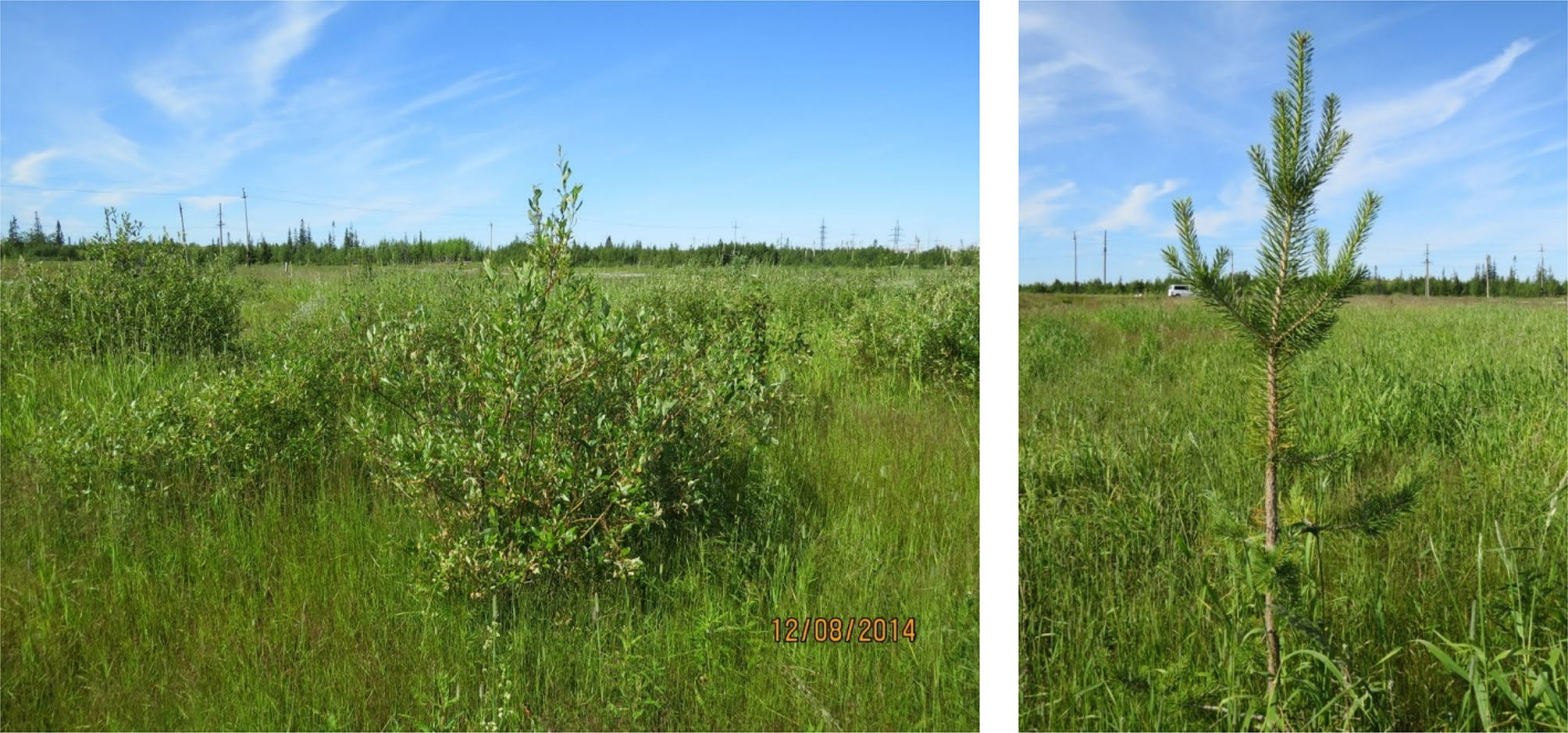
Presence of willow (*Salix glauca*) on plot 3 (left) and young pine (*Pinus sylvestris*) on plot 9 (right) in 2014.

In the year 2014, the C, S, and intermediate CSR-type species started playing an important role in the vegetation communities. These were mainly cereals (*Poa pratensis* (CSR) and *Calamagrostis purpurea* (C/CS)) and some species of the sedge family (*Eriophorum vaginatum* (S/CS), *Carex canescens* (−), *Carex rostrata* (CS), and *Carex limosa* (−)). In addition, the appearance of several willow species (*Salix caprea* (C/CS), *Salix lapponum* (−)) and single young trees (*Larix sibirica* (−), *Pinus Sylvestris* (−)*,* and *Picea obovate* (−)) was observed (Fig. 3).

Compared to 2006, by 2014 the total projective cover of vegetation had slightly decreased to 60–70%. By then, the number of species and the Shannon biodiversity index had also decreased for all plots, except for plot 9 (Table 3). The number of species increased from 19 (2006) to 22 (2014). The control plot (plot 2), where vegetation was completely absent in 2006, also showed an increase in the number of species, having up to 17 species of vascular plants in 2014. However, the 2014 projective cover of vegetation at the control plot was only 25–30% smaller than that at the other experimental plots. In the reference plant community, the number of species remained stable.

**Figure 4:**
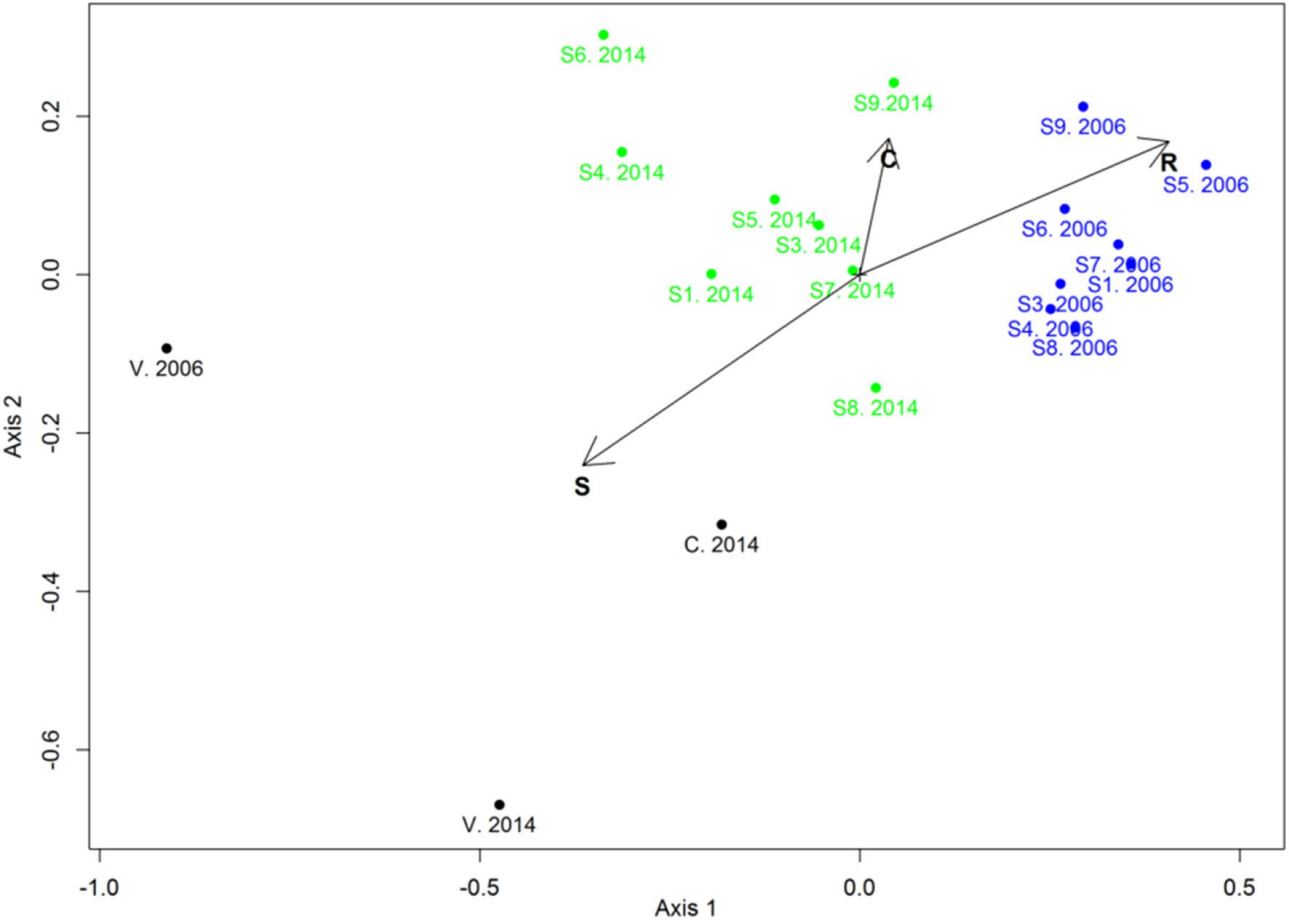
Non-metric multidimensional scaling (NMDS) ordination of relevés based on Bray–Curtis distance for experimental (S1 to S9), control (C), and reference (V) plots. Experimental plots from 2006 (blue) and 2014 (green). Control and reference plots 2014 (black).

The relevés analysis by NMDS ordination revealed the presence of two distinct groups (Fig. 4). All the experimental plots of 2006 were in the first group, while those of 2014 were in the second cluster. Reference and control plots lie separately on the NMDS diagram.

C, S, and R vectors indicate the correlation between NMDS axes and community-weighted mean (CWM) scores calculated for relevés.

**Figure 5:**
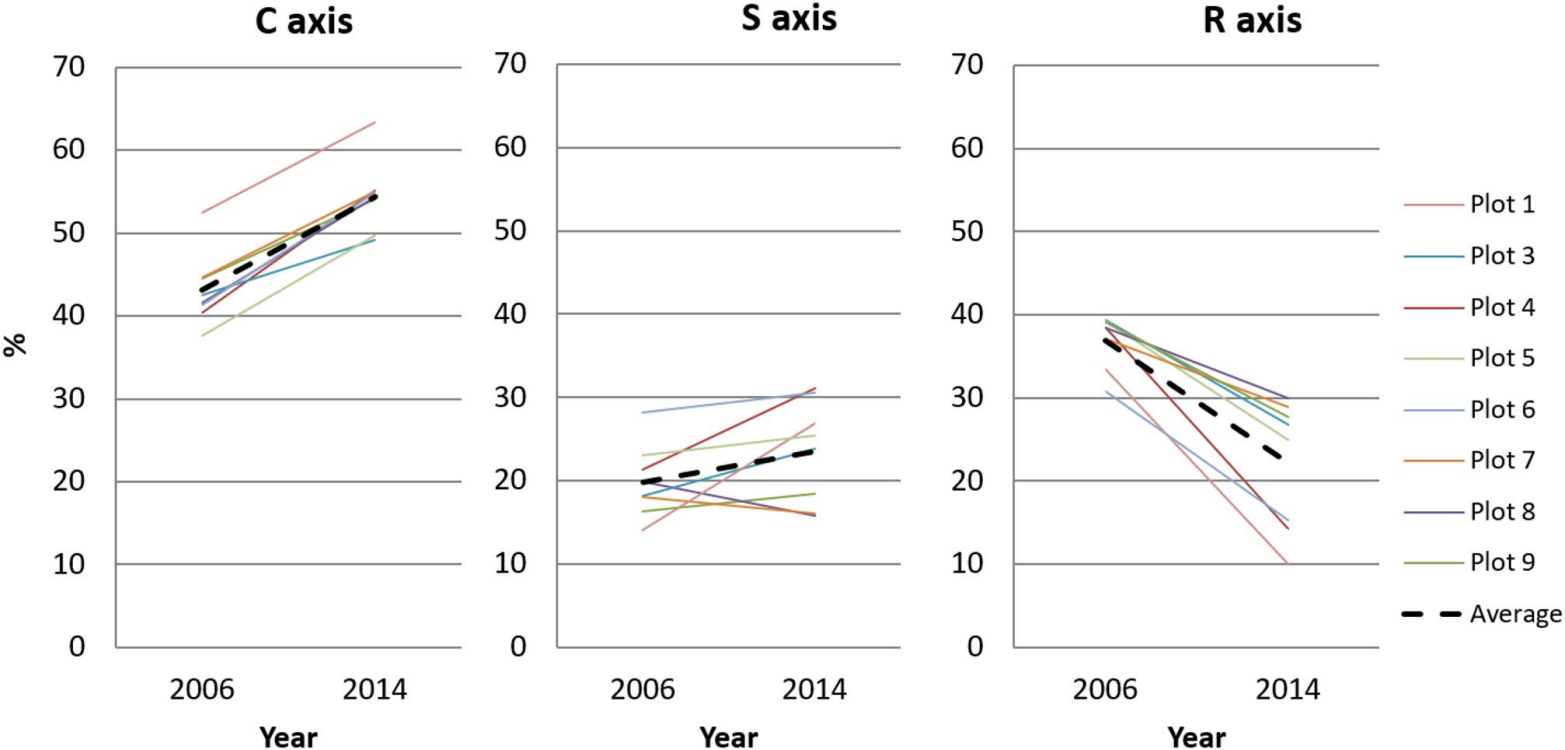
Changes in the community-weighted mean (CWM) C, S, and R scores at experimental plots. Dashed lines indicate averaged values.

Changes in the species composition and abundance over time led to significant changes in the average CWM scores along the C, S, and R axes (Table 4). The competitive (C) score increased from 37–45% in 2006 to 49–55% in 2014, and the corresponding ruderality (R) score decreased from 30–40% to 10–30% (Fig. 5). The average score along the stress tolerance (S) axis did not significantly change and was 18–25% for all experiments. It should be noted that all the values for the experimental plots are substantially less than those of the control (C—39%, S—51%, R—10%) and reference (C—29–36%, S—60–62%, R—4–9%) plots.

**Table 4:** Average competition–stress–ruderality (CSR) scores and biodiversity indices for experimental plots.

	Average values ± SD		Wilcoxon tests	
	2006	2014	W	p-value
C	43.5 ± 4.8	54.3 ± 4.1	2	< 0.001
S	18.8 ± 5.1	23.0 ± 6.1	24	0.158
R	37.7 ± 3.4	22.9 ± 7.2	81	< 0.001
Project cover	70.9 ± 13.4	65.0 ± 14.8	39	0.490
Number of species	17.8 ± 3.7	13.4 ± 5.3	48	0.101
Shannon index (H)	2.3 ± 0.7	1.9 ± 0.7	39.5	0.461

SD denotes standard deviation; C, S, and R are scores on the competitiveness, stress tolerance, and ruderality scales, respectively; Sample size n = 8.

**Figure 6:**
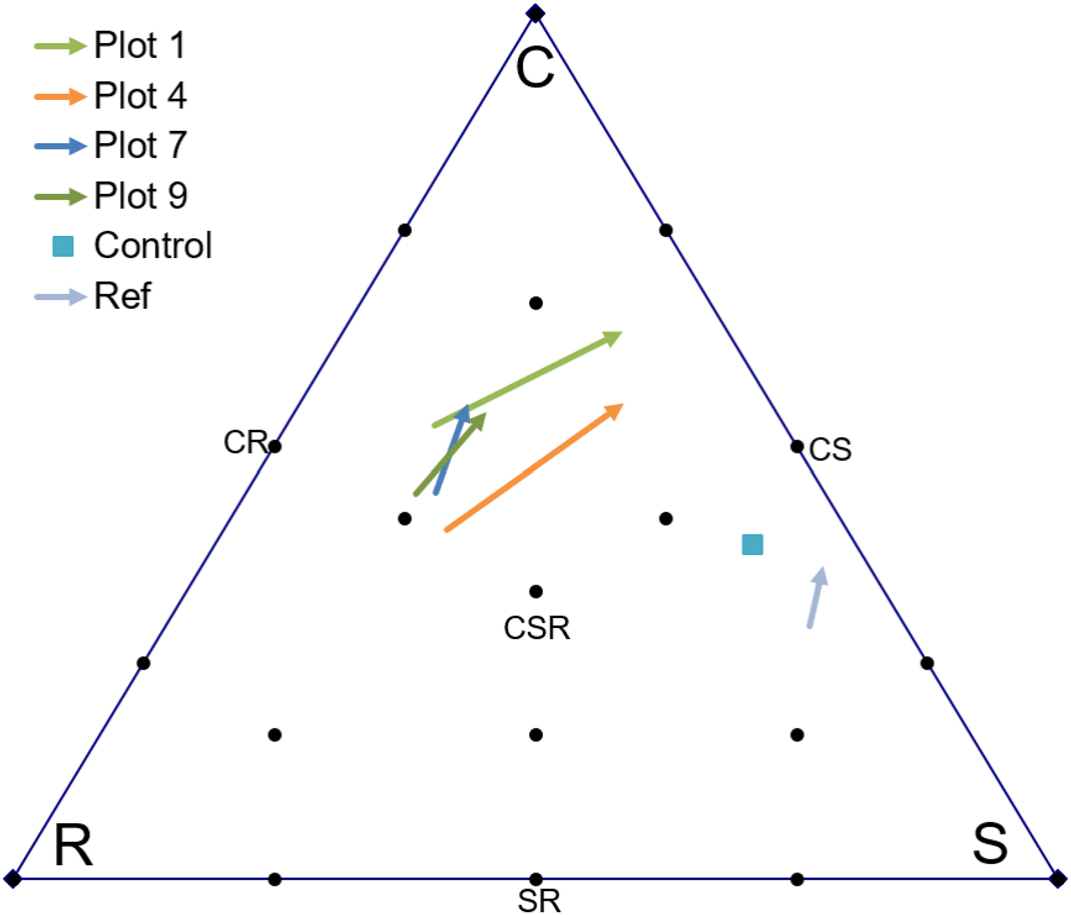
Trajectories of changes in competition–stress–ruderality (CSR) positions for experimental and reference plots. Only the plots with the two lowest and the two highest Euclidian distance (Table 3) are shown. Arrow: rear end corresponds to 2006 and tip to 2014. Blue square represents control plot in 2014. Ref represents reference plot.

For all plots, the individual analysis of the CWM changes revealed an approximately equal increase in the competition scores (10–20%) and a decrease in the ruderal scores (10–30%), as shown in Fig. 6. Along the stress tolerance axis, these changes are differently directed. Due to the increase in *Deschampsia cespitosa* (CS/CSR strategy) abundance and the appearance of *Eriophorum vaginatum* (S/CS strategy), the stress tolerance scores increased for most of the experimental plots. However, we observed a slight decrease in the stress tolerance scores for plots 7 and 8. This change was caused by a decrease in the *Phleum pratense* (CSR strategy) abundance.

The calculated Euclidean distance revealed the number of changes for all experimental plots, between 2006 and 2014, in CSR coordinates. The minimum difference, 13.4, was observed in plot 7 (agro-stimulation without biopreparation). The maximum changes, approximately 30, were observed in plots 1 and 4. For the reference plot, the difference was 8.8 (Table 3; Fig. 6).

An analysis of the trajectories in the CSR triangle reveals a slight converging of the experimental plots results with those of the control and reference territories (Fig. 6). It should be noted that the control plot has the closest position to the reference territory.

## Discussion

During this study, visible oil pollution manifestations disappeared at all test plots, except for plot 8, which contained the largest initial amount of oil. In the first period (2002–2006), almost all experimental plots (except control and plot 5) showed a statistically significant decrease in the oil content. However, in the second period (2006–2014), only the control plot and two out of the eight plots treated with biopreparation showed significant changes (Table 2).

A single use of biopreparation in 2002 encouraged the rapid development of bacteria which process oil degradation^39,63^. In the control plot, there was no such boost and hence, the oil decomposition rate was lower. Later, the bacterial count in the control plot increased naturally, which in turn increased the oil decomposition rate.

By 2014, the total oil reduction in the control plot was 51%, which was only slightly lower than some biopreparations. For example, the minimum decomposition rate of 54–55% was observed in plots 3 and 5. In contrast, the maximum oil decomposition rate of 90–92% was observed in plots 6 and 9 (Table 2). These plots were treated with the “Universal” (Institute of biology Komi Science Center) and “Roder” biopreparations (the Chemical Faculty of Moscow State University). However, it should be noted that by 2014, the residual oil contamination of all the plots did not return to the initial values and was higher than that of the reference plots.

The vegetation recovery rate varied widely and depended on the type of remediation technology used and the soil water content. By 2014, the projective cover of the vegetation of the biopreparation-treated variants was 60–70%. The control plot treated only by mechanical cleaning had only a 25–30% projective cover. In 2006, the average projective coverage at the experimental plots was slightly higher than that of 2014 and was 70–75% (Table 3). This species-abundance decrease was associated mainly with the water regime restoration outcome of the siltation of drainage channels, and an increase in the groundwater level.

Along with the decrease in projective cover, we observed a decline in the plant species number from 15–22 in 2006 to 6–17 in 2014. The exception was plot 9 (Table 3), where the total projective cover did not change, and the number of plant species increased.

By 2014, *Avena sativa* and *Trifolium pratense*, which were sown during phytoremediation, had disappeared. Oats (*Avena sativa*) is an annual species with exceptional seed regeneration. Under the condition of cold and short summer typical of forest-tundra, oats do not have the time to form seeds. Therefore, all observed seedlings were from seeds that did not germinate in the previous years. The depletion of the seed bank led to the complete disappearance of this species. Red clover (*Trifolium pratense*) is a perennial species which reproduces by seeds and vegetatively. Clover grows in this geographical area but under milder conditions, usually found in relief depressions or floodplains of large rivers. On the plateau, this species does not survive.

In addition, we observed that the ruderal species had almost completely disappeared. These species include the pioneer plants (annual and biennial), which first appeared on open ground due to rapid seed regeneration, and the perennial weeds, which reproduce mainly vegetatively. In 2006, there were 11 species with prevalent ruderal strategy (R, R/CR, or R/CSR). By 2014, there were only three of such species (Suppl. material). The presence of the ruderal species on the plots in 2006 could be attributed to the soil milling performed before the experiment. The vegetation cover was mechanically destroyed, and the restoration succession began anew, leading to the appearance of these species.

The ruderals were gradually replaced by species exhibiting competitive, stress-tolerant or intermediate strategies. This is the classic behavior of plant communities predicted by Grime’s theory^43^. Under productive ecological conditions, ruderal species are replaced by competitors, due to their ability to capture most of the available resources (water, light, mineral nutrients). In chronically unproductive habitats, ruderal species are replaced by stress-tolerant ones, which have a high survival rate in the long term.

In 2014, on the experimental plots, we observed the presence of the species which were used in remediation (*Agrostis gigantean*, *Deschampsia cespitosa*, *Phalaris arundinacea*, *Phleum pretense*) and the appearance of several new species: sedges (*Carex canescens*, *Carex limosa*, *Carex rostrata*), and cereals (*Calamagrostis purpurea*, *Poa pratensis*). These species were typical native flora, having competitor or stress-tolerant strategy, and showing high resistance to oil contamination^41,64,65^. Among the newly observed species, the most widespread (6 plots out of 9) was *Eriophorum vaginatum*. This species is reportedly tolerant of oil contamination^7,66,67^, at least in the northern regions.

A possible reason for the increase in the diversity of sedges family species was the waterlogging of experimental plots which reduced the total project cover and decreased the competition between species. Another possible advantage of sedges is their resistance to oil spills following their natural sustainability despite oxygen deficiency in their root layers^68^.

In 2014, the first trees and shrubs of the *Salix* genus appeared on the experimental plots (Fig. 3). Willows have good resistance to the negative effects of oil contamination. They grow quickly, have deep-rooting characteristics and are often used in phytoremediation^4,40,69,70^. The appearance of willows and trees indicated the restoration of the vegetation cover.

Another sign of restoration is the detection of the first mosses (*Calliergon giganteum* and *Polytrichum commune*). They covered only 5–10% of the study plots, which is less than their coverage in background communities. Mosses and lichens are known for being the most vulnerable to oil pollution^5,66^ and, therefore, their appearance indirectly indicated a restoration of the contaminated areas^71^.

The NMDS ordination (Fig. 4) revealed a pronounced gradient of the plant communities studied. The upper right corner shows the relevés of 2006. They are characterized by the prevalence of R-species. The relevés of 2014 display high C-scores and an intermediate position in the ordination space. The reference and control communities are dominated by S-species; they are located in the lower-left corner of the NMDS diagram. In other words, for all the experimental plots, we observed a clear shift from the prevalence of R-species towards the dominance of C and CS species. These changes are characteristic of abandoned areas^48^. The overgrowing occurs primarily due to competitive cereals (*Agrostis gigantea*, *Phalaris arundinacea*, *Deschampsia cespitosa,* and *Poa pratensis*). These species have good survival rates, produce relatively large biomass, and form a dense turf, impeding the introduction of typical bog and tundra species with S-strategy prevalent in the native vegetation.

At the control plot, where no biopreparation and plant seedling were used, we observed the typical tundra-bog S-species such as *Empetrum hermaphroditum, Eriophorum angustifolium, Eriophorum vaginatum, Vaccinium myrtillus, and Lycopodium clavatum*, as well as young birch, spruce, and willow species. The vegetation at this plot displayed a low projective cover and the latest overgrowing onset^63,72^. However, it had the closest position to the reference plots in terms of ordination (Fig. 4) and CSR scores (Fig. 6).

Phytoremediation employs plant species that display the best resistance to oil contamination, that have a high growth rate and high productivity, and can participate in the biological cleansing processes of the soil. Thus, sedges and cereals are the best candidates^29,64,65,71^ for heavily oil contaminated areas. Shrubs^40^, trees^24,73,74^, and crop plants^26,75^ are usually cultivated in areas with low oil concentration. Matching the plant species used for remediation with the natural local flora is not usually considered. For example, in this experiment, we used C-species cereals, which did not allow the native S-plants to populate the recovery area. This led to the replacement of natural vegetation with an intrazonal one. In addition, to restore soils, land melioration (partial drainage of wetlands) is often carried out. This leads to a change in the hydrological regime of the soils and, as a result, stimulates changes in the plant communities.

In our opinion, the leading indicator of the eligibility of different recovery methods should be the expected long-term impact on oil-contaminated areas. The main factors to take into account are the biological features of the plants, such as resistance to oil contamination and potential biomass productivity, as well as their affinity for the natural flora^42^. The restored community should resemble, as close as possible, the original communities located on the affected territory before the oil spills. It is preferable to use plant species from the local flora, such as mire sedges or willows. They have a fast growth rate and do not significantly change the plant communities’ appearance and physico-chemical properties.

## Conclusions

The remediation methods applied on the experimental plots reduced the oil content of the soil by 55–90% and allowed the development of productive plant communities. Despite the use of various methods of soil remediation and the amount of residual oil pollution, the vegetation recovery showed the same patterns over the twelve-year period. The vegetative projective cover in 2014 was approximately 50–70% and vegetation assemblages were also similar.

For all the plots, a significant increase in the number of competitive species and decrease in ruderal species was noticed. This indicated the clear shift of the communities from pioneer to stable state. The relatively small amount of stress-tolerant species and the resulting low scores along the S-axis indicate significant differences between the background vegetation (S-species prevalence) and the recovered plant communities (C-species dominance) at all experimental plots. Only the control plot (subjected only to mechanical cleaning; no plant seedlings and biopreparation) exhibited a close position to the reference territory in the CSR term. However, this plot displayed the lowest vegetation restoration rate; its projective cover of vegetation in 2014 was 2 to 2.5 times lower than those at the experimental plots.

Based on the theoretical principles of CSR theory, it can be assumed that the communities of the experimental plots will be relatively stable. The presence of a large number of competitor species (primarily cereals) hampers the growth of typical tundra and mire plant species at the remediated plots and prevents the restoration of background ecosystems. Employing only mechanical cleaning methods allowed the communities to recover a vegetation similar to the background vegetation; however, the recovery rate of this community was minimal.

In future studies, we plan to investigate whether the observed changes are stable in the long-term or whether the vegetation will return to its original state in a few years. Furthermore, it will be imperative to evaluate the differences in the rate of vegetation restoration of the treated and control plots.

## Supplementary Information

Supplementary Information

## References

[1] Liste H-H, Felgentreu D (2006). Crop growth, culturable bacteria, and degradation of petrol hydrocarbons (PHCs) in a long-term contaminated field soil. Appl. Soil. Ecol..

[2] Smith MJ, Flowers TH, Duncan HJ, Alder J (2006). Effects of polycyclic aromatic hydrocarbons on germination and subsequent growth of grasses and legumes in freshly contaminated soil and soil with aged PAHs residues. Environ. Pollut..

[3] Meudec A, Poupart N, Dussauze J, Deslandes E (2007). Relationship between heavy fuel oil phytotoxicity and polycyclic aromatic hydrocarbon contamination in *Salicornia fragilis*. Sci. Total Environ..

[4] Euliss K, Ho C-H, Schwab AP, Rock S, Banks MK (2008). Greenhouse and field assessment of phytoremediation for petroleum contaminants in a riparian zone. Bioresour. Technol..

[5] Hutchinson TC, Freedman W (1978). Effects of experimental crude oil spills on subarctic boreal forest vegetation near Norman Wells, N.W.T., Canada. Can. J. Bot..

[6] Lin Q, Mendelssohn IA (1998). The combined effects of phytoremediation and biostimulation in enhancing habitat restoration and oil degradation of petroleum contaminated wetlands. Ecol. Eng..

[7] Racine CH (1994). Long-term recovery of vegetation on two experimental crude oil spills in interior Alaska black spruce taiga. Can. J. Bot..

[8] Fatima K, Afzal M, Imran A, Khan QM (2015). Bacterial rhizosphere and endosphere populations associated with grasses and trees to be used for phytoremediation of crude oil contaminated soil. Bull. Environ. Contam. Toxicol..

[9] Hashmat AJ (2019). Characterization of hydrocarbon-degrading bacteria in constructed wetland microcosms used to treat crude oil polluted water. Bull. Environ. Contam. Toxicol..

[10] Khan FI, Husain T, Hejazi R (2004). An overview and analysis of site remediation technologies. J. Environ. Manag..

[11] Sarkar D, Ferguson M, Datta R, Birnbaum S (2005). Bioremediation of petroleum hydrocarbons in contaminated soils: comparison of biosolids addition, carbon supplementation, and monitored natural attenuation. Environ. Pollut..

[12] Gan S, Lau EV, Ng HK (2009). Remediation of soils contaminated with polycyclic aromatic hydrocarbons (PAHs). J. Hazard. Mater..

[13] Anchugova EM, Melekhina EN, Markarova MYu, Shchemelinina TN (2016). Approaches to the assessment of the efficiency of remediation of oil-polluted soils. Eurasian Soil Sci..

[14] Erkenova MI, Tolpeshta II, Trofimov SY, Aptikaev RS, Lazarev AS (2016). Changes of the content of oil products in the oil-polluted peat soil of a high-moor bog in a field experiment with application of lime and fertilizers. Eurasian Soil Sci..

[15] Sorkhoh NA (2011). Bioremediation of volatile oil hydrocarbons by epiphytic bacteria associated with American grass (*Cynodon* sp.) and broad bean (*Vicia faba*) leaves. Int. Biodeterior. Biodegrad..

[16] Roy AS (2014). Bioremediation potential of native hydrocarbon degrading bacterial strains in crude oil contaminated soil under microcosm study. Int. Biodeterior. Biodegrad..

[17] Cai B (2016). Comparison of phytoremediation, bioaugmentation and natural attenuation for remediating saline soil contaminated by heavy crude oil. Biochem. Eng. J..

[18] Murygina V, Gaydamaka S, Gladchenko M, Zubaydullin A (2016). Method of aerobic-anaerobic bioremediation of a raised bog in Western Siberia affected by old oil pollution. A pilot test. Int. Biodeterior. Biodegrad..

[19] Tahseen R (2016). Rhamnolipids and nutrients boost remediation of crude oil-contaminated soil by enhancing bacterial colonization and metabolic activities. Int. Biodeterior. Biodegrad..

[20] Baoune H (2019). Effectiveness of the Zea mays-Streptomyces association for the phytoremediation of petroleum hydrocarbons impacted soils. Ecotoxicol. Environ. Saf..

[21] Ra T, Zhao Y, Zheng M (2019). Comparative study on the petroleum crude oil degradation potential of microbes from petroleum-contaminated soil and non-contaminated soil. Int. J. Environ. Sci. Technol..

[22] Rajkumari J, Bhuyan B, Das N, Pandey P (2019). Environmental applications of microbial extremophiles in the degradation of petroleum hydrocarbons in extreme environments. Environ. Sustain..

[23] Newman LA, Reynolds CM (2004). Phytodegradation of organic compounds. Curr. Opin. Biotechnol..

[24] Unterbrunner R (2007). Plant and fertiliser effects on rhizodegradation of crude oil in two soils with different nutrient status. Plant Soil.

[25] Muratova AY, Dmitrieva TV, Panchenko LV, Turkovskaya OV (2008). Phytoremediation of oil-sludge-contaminated soil. Int. J. Phytorem..

[26] Shirdam R, Zand A, Bidhendi G, Mehrdadi N (2008). Phytoremediation of hydrocarbon-contaminated soils with emphasis on the effect of petroleum hydrocarbons on the growth of plant species. Phyto.

[27] Farias V (2009). Phytodegradation Potential of *Erythrina crista-galli* L., Fabaceae petroleum-contaminated soil. Appl. Biochem. Biotechnol..

[28] Peng S, Zhou Q, Cai Z, Zhang Z (2009). Phytoremediation of petroleum contaminated soils by *Mirabilis jalapa* L. in a greenhouse plot experiment. J. Hazard. Mater..

[29] Basumatary B, Saikia R, Bordoloi S, Das HC, Sarma HP (2012). Assessment of potential plant species for phytoremediation of hydrocarbon-contaminated areas of upper Assam, India. J. Chem. Technol. Biotechnol..

[30] Moubasher HA (2015). Phytoremediation of soils polluted with crude petroleum oil using Bassia scoparia and its associated rhizosphere microorganisms. Int. Biodeterior. Biodegrad..

[31] Fatima K, Imran A, Amin I, Khan QM, Afzal M (2016). Plant species affect colonization patterns and metabolic activity of associated endophytes during phytoremediation of crude oil-contaminated soil. Environ. Sci. Pollut. Res. Int..

[32] Khan S, Afzal M, Iqbal S, Khan QM (2013). Plant–bacteria partnerships for the remediation of hydrocarbon contaminated soils. Chemosphere.

[33] Yavari S, Malakahmad A, Sapari NB (2015). A review on phytoremediation of crude oil spills. Water Air Soil Pollut..

[34] Okoh E, Yelebe ZR, Oruabena B, Nelson ES, Indiamaowei OP (2020). Clean-up of crude oil-contaminated soils: bioremediation option. Int. J. Environ. Sci. Technol..

[35] Naeem, U. & Qazi, M. A. Leading edges in bioremediation technologies for removal of petroleum hydrocarbons. *Environ. Sci. Pollut. Res.* **27**, 27370–27382 (2019). 10.1007/s11356-019-06124-8 31392621

[36] Tyagi M, da Fonseca MMR, de Carvalho CCCR (2011). Bioaugmentation and biostimulation strategies to improve the effectiveness of bioremediation processes. Biodegradation.

[37] Fatima K, Imran A, Amin I, Khan QM, Afzal M (2018). Successful phytoremediation of crude-oil contaminated soil at an oil exploration and production company by plants-bacterial synergism. Int. J. Phytoremediat..

[38] Walker DA (1987). Cumulative impacts of oil fields on Northern Alaskan Landscapes. Science.

[39] Maganov RU, Markarova MY, Mulyak VV, Zagvozdkin VE, Zaikin IA (2006). Nature conservation management at the oil and gas companies. Part 1. Reclamation of oil-polluted lands in the Usinsky district of the Komi Republic.

[40] Vervaeke P (2003). Phytoremediation prospects of willow stands on contaminated sediment: a field trial. Environ. Pollut..

[41] Huang X-D, El-Alawi Y, Gurska J, Glick BR, Greenberg BM (2005). A multi-process phytoremediation system for decontamination of persistent total petroleum hydrocarbons (TPHs) from soils. Microchem. J..

[42] Robson DB, Knight JD, Farrell RE, Germida JJ (2004). Natural revegetation of hydrocarbon-contaminated soil in semi-arid grasslands. Can. J. Bot..

[43] Grime JP, Pierce S (2012). The evolutionary strategies that shape ecosystems.

[44] Ramenskiy LG (1935). On principal rules, basic concepts, and terms of land typology, geobotany, and ecology. Sov. Bot..

[45] Grime JP, Hodgson JG, Hunt R (1988). Comparative plant ecology: a functional approach to common British species.

[46] Thompson K, Boyle TJB, Boyle CEB (1994). Predicting the fate of temperate species in response to human disturbance and global change. Biodiversity, temperate ecosystems, and global change.

[47] Massant W, Godefroid S, Koedam N (2009). Clustering of plant life strategies on meso-scale. Plant Ecol..

[48] Novakovsky AB, Panyukov AN (2018). Analysis of successional dynamics of a sown meadow using Ramenskii–Grime’s System of ecological strategies. Russ. J. Ecol..

[49] Novakovskiy AB, Elsakov VV (2014). Hydrometeorological database (HMDB) for practical research in ecology. Data Sci. J..

[50] PND F 16.1: 2.21-98. Quantitative chemical analysis of soils. The method of measuring the mass fraction of oil products in soil and soil samples by the fluorimetric method on the Fluorat-02 fluid analyzer. https://www.russiangost.com/p-275219-fr131201213170.aspx

[51] Archegova, I. B., Markarova, M. Y. & Gromova, O. V. Method for reclaiming posttechnogenic lands and lands in remote districts of extreme North. US Patent RU2093974C1 (1997).

[52] Archegova, I. B., Markarova, M. Y. & Gromova, O. V. Method for producing granular fertilizing-seeding material. US Patent RU2099917C1 (1997).

[53] Murygina, V. P., Vojshvillo, N. E. & Kaljuzhnyj, S. V. Biological preparation ‘Roder’ for cleaning soils, soil grounds, sweet and mineralized waters to remove crude oil and petroleum products. US Patent RU2174496C2 (2001).

[54] Murygina VP, Markarova MY, Kalyuzhnyi SV (2005). Application of biopreparation “Rhoder” for remediation of oil polluted polar marshy wetlands in Komi Republic. Environ. Int..

[55] Lavorel S (2008). Assessing functional diversity in the field—methodology matters!. Funct. Ecol..

[56] Kindt, R. & Coe, R. Tree diversity analysis: a manual and software for common statistical methods for ecological and biodiversity studies (World Agroforestry Centre, Nairobi, 2006).

[57] Hodgson JG, Wilson PJ, Hunt R, Grime JP, Thompson K (1999). Allocating C-S-R plant functional types: a soft approach to a hard problem. Oikos.

[58] Pierce S, Brusa G, Vagge I, Cerabolini BEL (2013). Allocating CSR plant functional types: the use of leaf economics and size traits to classify woody and herbaceous vascular plants. Funct. Ecol..

[59] Magguran AE (2004). Measuring biological diversity.

[60] Peet RK (1974). The measurement of species diversity. Annu. Rev. Ecol. Syst..

[61] Kruskal JB (1964). Nonmetric multidimensional scaling: a numerical method. Psychometrika.

[62] McCune B, Grace JB (2002). Analysis of ecological communities.

[63] Melekhina EN, Markarova MYu, Shchemelinina TN, Anchugova EM, Kanev VA (2015). Secondary successions of biota in oil-polluted peat soil upon different biological remediation methods. Eurasian Soil Sci..

[64] Borowik A, Wyszkowska J, Gałązka A, Kucharski J (2019). Role of Festuca rubra and Festuca arundinacea in determinig the functional and genetic diversity of microorganisms and of the enzymatic activity in the soil polluted with diesel oil. Environ. Sci. Pollut. Res. Int..

[65] Wyszkowska J, Borowik A, Kucharski J (2019). The resistance of *Lolium perenne* L. × hybridum, *Poa pratensis*, *Festuca rubra*, *F. arundinacea*, *Phleum pratense* and *Dactylis glomerata* to soil pollution by diesel oil and petroleum. Plant Soil Environ..

[66] Freedman W, Hutchinson T (1976). Physical and biological effects of experimental crude-oil spills on Low Arctic Tundra in Vicinity of Tuktoyaktuk, Nwt, Canada. Can. J. Bot.-Rev. Can. Bot..

[67] Kazantseva MN (2011). The effect of oil extraction on ground cover of West Siberian taiga forests. Contemp. Probl. Ecol..

[68] Lapshina ED, Bleuten W (1999). Types of deterioration and self-restoration of vegetation of olygotrophic bogs in oil-production areas of Tomsk province, Krylovia. Siberian Bot. J..

[69] Cook RL, Landmeyer JE, Atkinson B, Messier J-P, Nichols EG (2010). Field note: successful establishment of a phytoremediation system at a petroleum hydrocarbon contaminated shallow aquifer: trends, trials, and tribulations. Int. J. Phytorem..

[70] Nichols EG (2014). Phytoremediation of a petroleum-hydrocarbon contaminated shallow aquifer in Elizabeth City, North Carolina, USA. Remediat. J..

[71] Seburn DC, Kershaw GP, Kershaw LJ (1996). Vegetation response to a subsurface crude oil spill on a subarctic right-of-way, Tulita (Fort Norman), Northwest Territories, Canada. Arctic.

[72] Melekhina EN, Markarova MYU, Anchugova EM, Shchemelinina TN, Kanev VA (2016). The efficiency assessment of oil polluted soil recultivation methods. Bull. Komi Sci. Center Ural Branch of RAS.

[73] Ma X, Burken JG (2002). VOCs fate and partitioning in vegetation: use of tree cores in groundwater analysis. Environ. Sci. Technol..

[74] Haroni NN, Badehian Z, Zarafshar M, Bazot S (2019). The effect of oil sludge contamination on morphological and physiological characteristics of some tree species. Ecotoxicology.

[75] Banks MK, Kulakow P, Schwab AP, Chen Z, Rathbone K (2003). Degradation of crude oil in the rhizosphere of sorghum bicolor. Int. J. Phytorem..

